# The Operative Treatment of Retro-Displacements of the Uterus1Read before the Buffalo Obstetrical Society.

**Published:** 1892-05

**Authors:** C. C. Frederick

**Affiliations:** Adjunct Professor of Obstetrics, Medical Department, Niagara University; Obstetrician and Gynecologist Buffalo Woman’s Hospital; 64 Richmond Avenue


Buffalo Medical and Surgical Journal

VOL. XXXI. MAY, 1892. No. 10.

### Original Communications.

### THE OPERATIVE TREATMENT OF RETRO-DISPLACEMENTS OF THE UTERUS.[1]

BY C. C. FREDERICK, B. S., M. D.,

Adjunct Professor of Obstetrics, Medical Department, Niagara University; Obstetrician and Gynecologist Buffalo Woman's Hospital.

IT IS but a few years ago that most of the various ills of woman were ascribed to a displaced uterus. Fortunately a great deal of this belief has given place to a more correct pathology and a consequent more correct treatment. One of the malpositions, viz., retro-displacements of the uterus, none of you will deny, holds an important position in its influence on the health and well-being of women. In support of this statement could be cited the histories of many cases in which immediate or gradual relief to local and constitutional symptoms, has followed the replacement of a retroverted uterus and the introduction of a properly fitting pessary.

The majority of women having retroversion have also uterine hypertrophy, catarrh, and sterility. If conception does occur, the retro-displacement is frequently the cause of much trouble in the early months of pregnancy, such as excessive vomiting, frequent and painful micturition, polyuria, and abortion. I have seen complete retention of urine at the third and fourth months of pregnancy, due to this displacement. Today I have been obliged to induce abortion, the patient being two and one-half months pregnant. She had a retroverted adherent uterus which could not be replaced; the vomiting was excessive and exhausting nearly to the point of collapse. Before she had the pelvic peritonitis which bound down her uterus and its appendages, she carried her children with less nausea than most women experience. We, therefore, probably, do not over-estimate its importance in the causation of distressing symptoms.

1. Read before the Buffalo Obstetrical Society.

Backward displacements requiring operative treatment fall into either of the two classes:

1. Those which cannot be held up by pessaries when replaced.
2. Those which cannot be replaced because either the uterus or the appendages are adherent from previous pelvic inflammation

To the first class belong those associated with or caused by lacerations of either cervix or perineum, or both; in which case pessaries will probably fall out of the vagina, or will sag to a sufficient degree to allow the uterus to tip backward. Repair of the lacerations, which corrects the subinvolution of the uterus and restores the retentive power of the vagina, makes it possible to correct the displacement, unless there be a prolapse of one or both of the ovaries. A prolapsed ovary, whether it be adherent or not, if it is not possible to get it out of the way so as to avoid its being pressed upon by the pessary, is an effective barrier to the efficient use of this support. Therefore, any retroversion causing distress, if associated with lacerations of the cervix or perineum, should first be subjected to repair of the lacerations. If lacerations complicate those cases in which there has been pelvic inflammations with adhesions, the repair of the lacerations may be one step in the right direction, but this will not free the uterus and allow of its restoration to normal position. To do this, some operative method must be sought.

Since abdominal surgery has reached its present stage of development, there have been devised several methods of treating successfully all those cases which formerly were irremediable.

The first was Alexander's operation, which, as you know, consists in shortening the round ligaments, by cutting down upon them as they emerge from the inguinal canal, and pulling them out far enough to raise the uterus to the required position, and stitching them into the wound. Alexander's operation has never met with general favor. It is applicable to but a limited class of cases. Patients subjected to it have been reported, in many instances, as suffering as much pain after operation as before, from the pulling in the scar. I have now under my care a young woman who has had this operation done by a Western surgeon with no benefit, as she is obliged to wear a retroversion pessary. Without the pessary, she complains of pain throughout the pelvis, probably caused by traction on the round ligaments.

The operations which have met with most success are some forms of ventral fixation of the uterus, or shortening of the round ligaments by laparatomy. There are several methods of ventral fixation, dependent upon the condition of the uterus and its appendages. If there is no disease of the tubes or ovaries, nothing to remove, the fundus uteri may be denuded of epithelium by scraping it with a scalpel, and two or three of the stitches in the lower end of the incision passed through the fundus, thus drawing it up against the abdominal wall, where it will adhere and remain after the stitches are removed.

The round ligaments may be shortened by Wylie's method. The uterus is lifted into normal position, and the slack in the round ligaments taken up by lapping a fold of each upon itself and stitching it. Dr. A. P. Dudley shortens the round ligament by passing a stitch beneath it at any distance from the uterus estimated as necessary. He then passes the thread through the fundus, which has previously been denuded of a small patch of epithelium, thence the thread is carried beneath the opposite round ligament, at the same distance from the uterus as the first stitch. When the silk is tied, the ligaments are shortened and approximated to the fundus, where they adhere. They also have a longer leverage upon the uterus than before.

Dr. Polk and Dr. Howard Kelly have each reported modifications of the intra-abdominal fixation methods which they have used.

If the appendages are removed, their stumps may be sewed to the abdominal wall, or a stitch passed through the fundus, as before described. The latter method I have done. I have had no experience with Dr. Dudley's method, but, on theoretical grounds, and from the reported experiences of others, I believe it to be the best method yet devised.

A uterus found retroverted during laparatomy should always be brought forward and secured by some one of these various methods. Even when the tubes and ovaries are removed, a retroverted uterus may give trouble, although we would expect that atrophy of the organ following this operation would forever prevent its causing any further disturbance. The experience of the oldest operators will coincide with this statement.

While in Europe recently, I saw several methods, both intra-abdominal and vaginal, in course of trial. Most of the vaginal methods seemed to me unsurgical, several of them requiring the peritoneal cavity to be opened through Douglas's cul-de-sac, to break up adhesions, before resorting to the fixation. The intra-abdominal plan is most direct and surgical, and such seemed to be the opinion of all the best operators whom I saw.

The question naturally arises, What will be the effect upon a subsequent pregnancy if the uterus is sewed to the anterior abdominal wall ? The same question cannot be raised when only intraperitoneal shortening of the round ligaments has been done. Ventral fixation is more used in Europe than any other method. Martin, Leopold, and Olshausen told me of pregnancies going on subsequent to the operation as though no fixation had been made. Furthermore, Leopold told me that he had examined three women who had borne children after operation, and the uterus in each case was in position. This same fact is observed in all Cesarean sections done more than once upon the same woman. I saw Leopold do one Cesarean section, it being the second the woman had had, and another which was the third the woman had undergone. In both of these there was extensive adhesions all along the line of the old uterine and abdominal incisions, and they had not interfered in the least with the course of pregnancy.

The points to which I wish to call your especial attention are :

1. That retroversions caused by or aggravated by accompanying lacerations of cervix or perineum, should have the lacerations closed to insure relief and reposition.

2. A uterus or appendage bound down posteriorly cannot be raised, nor the adhesions broken up manually through the rectum or vagina, unless they be very recent or slight. They must be broken up by laparatomy and the uterus restored and retained in position by some one of the many plans now used.

3. No malposition of the uterus needs operative treatment unless it renders the woman sterile or causes her physical suffering or inconvenience. Those whose condition demands relief should be given the benefit of some of these various methods which have been proven safe and efficient.

64 Richmond Avenue.

---

Lactation and Pregnancy.—Dr. Lewellyn Eliot, of this city, discussed Dr. Walsh's paper and spoke of the "popular fallacy" among women, that so long as they are nursing a baby there is no danger of their becoming pregnant. Dr. Eliot also stated that the amount of nourishment in breast-milk, after the tenth month, was no greater than in ordinary beef-tea.—*National Medical Review.*

